# Elevated perceived threat is associated with reduced hippocampal volume in combat veterans

**DOI:** 10.1038/s41598-019-51533-x

**Published:** 2019-10-17

**Authors:** Daniel W. Grupe, Benjamin A. Hushek, Kaley Davis, Andrew J. Schoen, Joseph Wielgosz, Jack B. Nitschke, Richard J. Davidson

**Affiliations:** 1 Center for Healthy Minds, University of Wisconsin-Madison, Madison, WI USA; 2 The Waisman Laboratory for Brain Imaging and Behavior, University of Wisconsin-Madison, Madison, WI USA; 3 Department of Computer Science, University of Wisconsin-Madison, Madison, WI USA; 4 Department of Psychiatry, University of Wisconsin-Madison, Madison, WI USA; 5 Department of Psychology, University of Wisconsin-Madison, Madison, WI USA; 6 Sierra Pacific MIRECC, VA Palo Alto Healthcare System, Palo Alto, California USA; 7 Department of Psychiatry and Behavioral Sciences, Stanford University, Palo Alto, California USA

**Keywords:** Emotion, Stress and resilience, Post-traumatic stress disorder

## Abstract

Reduced hippocampal volume is frequently observed in posttraumatic stress disorder (PTSD), but the psychological processes associated with these alterations remain unclear. Given hippocampal involvement in memory and contextual representations of threat, we investigated relationships between retrospectively reported combat exposure, perceived threat, and hippocampal volume in trauma-exposed veterans. T1-weighted anatomical MRI scans were obtained from 56 veterans (4 women, 52 men; 39 with elevated PTSD symptoms, “PTSS” group) and hippocampal volume was estimated using automatic segmentation tools in FreeSurfer. Hippocampal volume was regressed on self-reported perceived threat from the Deployment Risk and Resilience Inventory, and combat exposure from the Combat Exposure Scale. As a secondary analysis, hippocampal volume was regressed on Clinician-Administered PTSD Scale (CAPS) symptoms. In veterans with elevated PTSD symptoms, hippocampal volume was inversely related to perceived threat while deployed while controlling for self-reported combat exposure. Hippocampal volume was also inversely correlated with avoidance/numbing CAPS symptoms. Future research should clarify the temporal milieu of these effects and investigate whether individual differences in hippocampal structure and function contribute to heightened threat appraisal at the time of trauma vs. subsequently elevated appraisals of traumatic events.

## Introduction

Reduced hippocampal volume is consistently observed in posttraumatic stress disorder (PTSD), with meta-analyses revealing these reductions across different trauma types and demographic groups^1–3^. The magnitude of this reduction, however, is quite modest. The largest study of subcortical structures in PTSD – a retrospective multi-site study consisting of nearly 1,900 participants – revealed an effect size of *d* = 0.17 for participants with PTSD vs. trauma-exposed controls^4^. Exposure to trauma alone, even in the absence of PTSD pathology, can be associated with volumetric reductions, as hippocampal volume in trauma-exposed controls falls between that of individuals with PTSD and unexposed controls^1,2^. These findings of modest effect sizes and volumetric differences in the absence of PTSD symptoms highlight the need for updated models of what is reflected in post-traumatic structural alterations to the hippocampus.

A separate line of research has investigated psychological and psychosocial risk factors to explain why different individuals exposed to similar traumatic events experience divergent long-term trajectories^5^. This question, however, may be based on a faulty premise: just because two individuals are exposed to the same external circumstances does not mean that they experienced the “same” trauma. The interpretation and meaning of these traumatic experiences will vary widely across individuals based on biological and psychological predispositions, past experiences, and current environmental factors. Indeed, subjectively *perceived threat* – fear or worry about one’s safety and well-being during and after exposure to trauma – is one of the best predictors of PTSD^5–7^ and other mood and anxiety disorders^8,9^, and mediates the relationship between combat exposure and PTSD symptoms in multiple veteran samples^10–12^. These relationships between subjective threat appraisals and the emergence of psychopathology underscore the importance of identifying neurobiological mechanisms of this psychological characteristic.

The hippocampus is a prime candidate region that may be related to subjectively perceived threat, due to its central role in the contextual processing of threat^13^ and the aforementioned evidence for structural alterations to the hippocampus following trauma exposure^1,2,14^. Two studies of healthy, older adults^15,16^ identified an inverse relationship between hippocampal volume and the related construct of perceived stress, the degree to which individuals appraise daily life events as being stressful, overwhelming, and uncontrollable. However, no studies to our knowledge have directly examined the relationship between perceived threat following combat trauma and hippocampus structure.

Another region to consider in relation to perceived threat is the amygdala, which appears to show reduced volume in PTSD based on meta-analytic and large-scale case-control studies^1,17^. A previous study in soldiers deployed to Afghanistan found that increased perceived threat while deployed – but not self-reported combat exposure – was associated with increases in pre- to post-deployment functional connectivity in a threat-responsive circuit involving the amygdala and dorsal anterior cingulate cortex^18^ that persisted approximately 16 months post-deployment^19^.

An important consideration in studying perceived threat in combat-exposed individuals is that deployment environments can be associated with objectively high levels of threat, in which case extreme levels of perceived threat may reflect contextually appropriate, adaptive responses. Whereas increased attentional biases toward threat in new military recruits predict the eventual development of PTSD during a safe baseline period, the opposite is true immediately prior to and during deployment, when increased threat avoidance is associated with later PTSD^20,21^. It is not the case that particular behavioral profiles are universally adaptive or maladaptive; rather, a defining characteristic of maladaptive threat responding is incongruence between a specific context or environment and one’s response^22^, making it important to consider the role of both trauma exposure and perceived threat on long-term outcomes. An important and unanswered question is whether post-combat alterations to the hippocampus or amygdala are associated with combat exposure, perceived threat during deployment, or both.

To that end, in a sample of 56 combat-exposed veterans, we investigated relationships between retrospective self-reports of perceived threat and combat exposure on the one hand and volume of the hippocampus and amygdala on the other. Within this sample, we focused on a group of 39 veterans with elevated PTSD symptoms, as this group had elevated levels of perceived threat and qualitatively different relationships between perceived threat and combat exposure than did asymptomatic veterans. We tested the hypothesis that greater perceived threat would be associated with reductions in hippocampal and/or amygdalar volume while controlling for self-reported combat exposure. As a secondary analysis, we also investigated volume of these structures as a function of PTSD symptom severity.

## Methods

### Participants

We recruited veterans of Operation Enduring Freedom/Operation Iraqi Freedom through community and online advertisements and in collaboration with veterans’ organizations, the Wisconsin National Guard, and the Madison Veterans Affairs Hospital. Following complete study description, written informed consent was obtained. All experimental procedures were approved by the University of Wisconsin-Madison Health Sciences IRB and methods were carried out in accordance with all relevant guidelines and regulations.

Following informed consent, a team of clinically trained interviewers administered the Clinician-Administered PTSD Scale for DSM-IV (CAPS)^23^ and Structured Clinical Interview for DSM-IV (SCID)^24^ with supervision from a licensed clinical psychologist (JBN). Exclusionary conditions included substance dependence within the past 3 months and lifetime bipolar, psychotic, or cognitive disorders.

At the time of enrollment, participants were assigned either to a combat-exposed control (CEC) group or a posttraumatic stress symptoms (PTSS) group (Table 1). Participants in the CEC group had no current Axis I disorder and very low PTSD symptoms (CAPS scores <10) and did not meet diagnostic criteria for any CAPS subscales. Participants in the PTSS group had PTSD symptoms occurring at least monthly with moderate intensity and CAPS scores ≥20 and met diagnostic criteria for at least 1 of 3 CAPS subscales. Subgroup criteria were determined in advance of statistical analyses to differentiate veterans with clinically significant symptom burden from those without.

**Table 1:** Demographic, clinical, and self-report data for the posttraumatic stress symptoms (PTSS) and combat-exposed control (CEC) groups.

	PTSS (N = 39)		CEC (N = 17)		Independent samples *t* test	
	Mean	SD	Mean	SD	*t*(54)	*p* value
Age	30.6	6.6	31.0	6.4	−0.20	0.84
Years since Deployment	4.9	2.7	5.6	3.0	−0.85^a^	0.40
CAPS Total	49.1	19.5	3.5	2.6	9.55	<0.001
CAPS B (Re-experiencing)	11.9	7.8	0.2	1.0	6.06	<0.001
CAPS C (Numbing/Avoidance)	17.4	9.9	0	0	7.19	<0.001
CAPS D (Hyperarousal)	19.8	7.2	3.2	2.7	9.24	<0.001
Combat Exposure Scale	20.6	8.8	16.1	9.0	1.76	0.14
DRRI: Perceived Threat	50.6	8.0	40.8	10.5	3.82	<0.001
Beck Depression Inventory	27.7	8.9	17.5	6.3	4.32	<0.001
Beck Anxiety Inventory	37.3	10.2	24.5	3.9	5.00	<0.001
Penn State Worry Questionnaire	47.5	17.0	31.8	12.6	3.44	0.0011

NOTES: ^a^One subject in the PTSS group was missing data for Years since Deployment. CAPS = Clinician-Administered PTSD Scale; DRRI = Deployment Risk and Resilience Inventory.

58 veterans (4 women, 54 men) met eligibility criteria and were enrolled. Data from 2 participants were excluded due to excessive motion that prevented accurate delineation of white/gray matter boundaries. The final sample consisted of 39 PTSS subjects and 17 veterans enrolled in the CEC group. Due to the wide range of variability in PTSD symptoms and consistent with dimensional approaches to investigating psychopathology^25^, we investigated subcortical structural volume as a function of continuous variability in the independent variables of interest (e.g., PTSD symptoms, perceived threat, and combat exposure). Primary analyses were restricted to the 39 PTSS subjects due to group differences in perceived threat as well as qualitatively different relationships between perceived threat and combat exposure between groups (see “Results: Clinical and self-report measures”).

Current treatment with psychotropic medications (other than benzodiazepines or beta-blockers) or maintenance psychotherapy was permitted if treatment was stable for 8 weeks. Psychotropic medication use included monoamine reuptake inhibitors (10/39 PTSS group, 12/56 total), tricyclic/tetracyclic antidepressants (2/39, 2/56), atypical antidepressants (1/39, 1/56), anxiolytics (buspirone; 2/39, 2/56), opioid pain medications (3/39, 3/56), sleep aids (zolpidem; 1/39, 2/56), anticonvulsants (Lamotrigine; 1/39, 1/56), and Prazosin (1/39, 1/56). In total, 13/39 participants in the PTSS group and 15/56 in the full sample were on a stable course of one or more psychotropic medications. In addition, 5/56 participants (all in the PTSS group) were receiving regular counseling or psychotherapy, 3 of whom were also receiving psychotropic medication.

We previously reported on relationships between PTSD symptoms and fMRI activation in two publications using overlapping samples^26,27^.

### Data collection

In a pre-MRI visit, participants completed self-report measures including the Combat Exposure Scale (CES)^28^ and subscales from the Deployment Risk and Resiliency Inventory (DRRI)^5^. The CES is a 7-item Likert scale assessing the frequency of different wartime stressors (example items: “Did you ever go on combat patrols or have other dangerous duty?”, “Were you ever under enemy fire?”). Internal consistency in our sample was in the fair to good range (PTSS group: α = 0.79, full sample α = 0.80). The DRRI includes 17 scales characterizing environmental, psychosocial, and psychological factors before, during, and after deployment. Among these scales is “perceived threat” (or “combat concerns”), a 15-item Likert scale reflecting veterans’ cognitive or subjective appraisals of combat-related danger (example items: “I thought I would never survive”, “I was concerned that my unit would be attacked by the enemy”). Internal consistency in our sample was in the fair to good range (PTSS group: α = 0.73, full sample α = 0.80). Other self-report measures of potential overlap with the construct of perceived threat were also collected: the Beck Anxiety Inventory^29^, Beck Depression Inventory^30^, and Penn State Worry Questionnaire^31^.

Participants took part in an MRI scan within the subsequent 40 days. MRI data were collected on a 3T X750 GE Discovery scanner using an 8-channel head coil and ASSET parallel imaging with an acceleration factor of 2. Brain structure was assessed through the collection of T1-weighted anatomical images with 1-mm isotropic voxels (“BRAVO” sequence, TR = 8.16, TE = 3.18, flip angle = 12°, field of view = 256 mm, 256 × 256 matrix, 156 axial slices). Self-reported PTSD symptoms were assessed on the day of the MRI scan using the PTSD Checklist-Military Version (PCL-M)^32^.

### Structural MRI processing

Cortical reconstruction and volumetric segmentation were performed using the FreeSurfer image analysis suite (stable release version 5.3.0; http://surfer.nmr.mgh.harvard.edu/). Processing included motion correction, skull removal, intensity normalization, registration, segmentation of subcortical white and deep gray matter structures, white matter and pial surface tessellation, and cortical surface parcellation. Segmentation quality was visually assessed and manually edited as necessary (http://freesurfer.net/fswiki/Edits). Automated segmentation of the bilateral hippocampus and amygdala was conducted for each subject, a procedure that compares favorably with labor-intensive manual segmentation^33^.

Voxelwise brain morphometry (VBM) analyses were conducted using FSL-VBM^34^ (http://fsl.fmrib.ox.ac.uk/fsl/fslwiki/FSLVBM). Structural images were brain-extracted using BET and segmented into white and gray matter. Gray matter images were registered to the MNI-152 standard space template using non-linear registration (FNIRT), and the resulting images were averaged and flipped along the x-axis to create a left-right symmetric, study-specific gray matter template. All subject-space gray matter images were non-linearly registered to this study-specific template and “modulated” to correct for local expansion (or contraction) due to the non-linear component of the spatial transformation, by multiplying each voxel of the registered gray matter image by the Jacobian of the warp field. The modulated gray matter images were then smoothed with an isotropic Gaussian kernel with a sigma of 3 mm.

### Data analysis

Multiple linear regression analyses were conducted in R Version 3.2.2 and always included a covariate of Gender unless otherwise indicated. Within the PTSS group, we regressed total hippocampal/amygdalar volume on combat exposure and perceived threat scores separately. We also conducted a simultaneous multiple regression of hippocampal volume on combat exposure and perceived threat scores. As a control analysis, we repeated volumetric analyses while including as a covariate current psychotropic medication use or stable psychotherapy (as detailed above, 15/39 participants in the PTSS group and 17/56 participants in the full sample were on a stable course of psychotropic medications and/or psychotherapy). As secondary analyses, we regressed hippocampal and amygdalar volume on total CAPS symptoms in an effort to replicate previously observed volumetric reductions, and on each CAPS subscale to explore relationships with specific symptom clusters. While the gold-standard, clinician-administered CAPS served as the primary PTSD symptom measure of interest, we conducted secondary analyses with self-reported PCL symptoms.

For VBM data, confirmatory small-volume-corrected analyses were conducted within the anatomically constrained amygdala and hippocampus, which were defined by thresholding Harvard-Oxford amygdala and hippocampus ROIs bilaterally at a 25% probability threshold and then combining these into a single ROI^35^. Spatially smoothed gray matter maps were regressed on DRRI-perceived threat and Combat Exposure Scale scores (separately and in the same model). Resulting statistical maps for these analyses were corrected for multiple comparisons at a threshold of *p* < 0.05 using permutation-based non-parametric testing (FSL’s randomise). We also conducted an exploratory search for whole-brain morphometric changes associated with each of these variables of interest using a threshold of two-tailed *p* < 0.05. Non-thresholded statistical maps are available at https://neurovault.org/collections/4497/.

## Results

### Clinical and self-report measures

Demographics and symptom data are provided in Table 1. The PTSS group had higher levels of PTSD, anxiety, depression, and worry scores. The groups did not differ on self-reported combat exposure on the CES, but the PTSS group reported elevated DRRI perceived threat.

**Figure 1:**
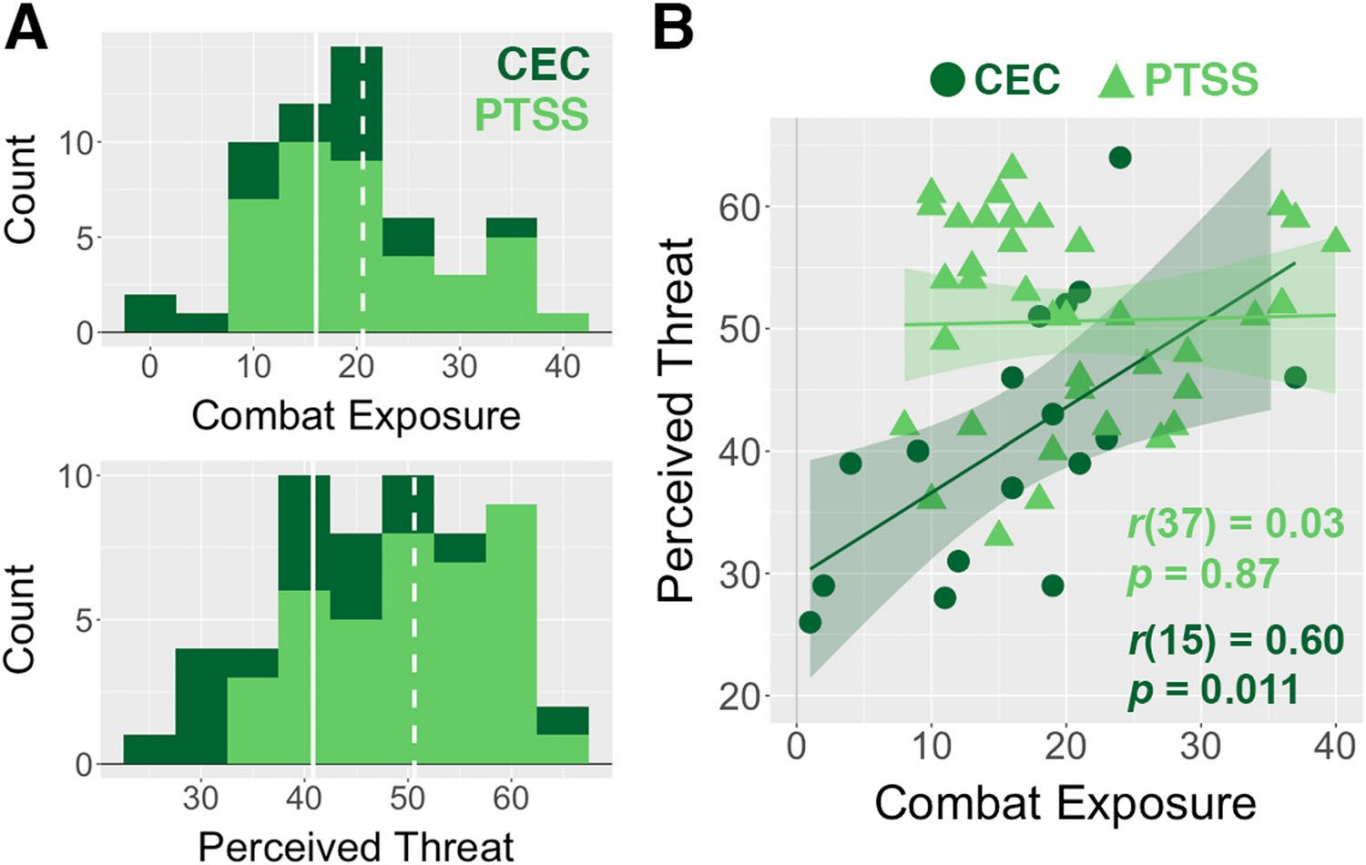
(**A**) Stacked histograms show the distributions of self-reported combat exposure and perceived threat for the posttraumatic stress symptoms (PTSS; light green) and combat-exposed control (CEC; dark green) groups. Relative to the CEC group, the PTSS group had elevated perceived threat (*t*(54) = 3.82, *p* < 0.001) and a trend toward greater self-reported combat exposure (*t*(54) = 1.76, *p* = 0.084). The solid and dashed lines indicate mean values for the CEC and PTSS groups, respectively. (**B**) Individuals in the CEC group showed a strong and positive correlation between combat experience and perceived threat, whereas this relationship was absent for individuals in the PTSS group. Shaded areas indicate 95% confidence intervals.

Consistent with previous research^9,11^, the combat-exposed control group showed a robust linear relationship between combat exposure and perceived threat (*r*(15) = 0.60, *p* = 0.011). This pattern was notably absent in the PTSS group (*r*(37) = 0.03, *p* = 0.87), consistent with a previous study in Dutch combat veterans^18^ (significant difference in correlation between groups, Fisher’s *Z* = 2.11, *p* = 0.035; Fig. 1B).

In the PTSS group, perceived threat was positively correlated with avoidance/numbing CAPS symptoms, and greater combat exposure was correlated with elevated re-experiencing CAPS symptoms (Table S1). Participants in the control group showed no associations between CAPS symptoms and either measure (Table S1), likely reflecting the small sample size and truncated range of CAPS scores. For the PTSS group, perceived threat showed robust positive correlations with depression, anxiety, and worry symptoms, whereas combat exposure was not reliably associated with these symptoms (Table S2). In the control group, perceived threat showed similar magnitude (though not significant) associations with depression, anxiety, and worry, whereas combat exposure was not associated with these symptoms.

In sum, analysis of self-report data revealed that the PTSS group had elevated levels of perceived threat relative to the control group and failed to demonstrate a presumably normative relationship between perceived threat and combat exposure. Because the PTSS group seemed to represent a distinct population from combat-exposed controls with regard to the perceived threat and combat exposure variables of interest, we focused subcortical volumetric analyses on participants in the PTSS group only (analogous analyses for the control group are provided in Table S3).

### Elevated perceived threat is associated with smaller hippocampal volume

Hippocampal and amygdalar volumes for the PTSS group (and, for reference, the combat-exposed control group) are provided in Table S4. Within the PTSS group, women had significantly smaller hippocampi than men (7489 mm^3^ vs 8874 mm^3^; *t*(37) = 3.04, *d* = 1.82, *p* = 0.0044). The analogous test for amygdala size was not significant, although a large effect size was estimated for this test (3352 mm^3^ vs 3689 mm^3^; *t*(37) = 1.46, *d* = 0.88, *p* = 0.15). We thus included gender as a covariate in all structural analyses (results were similar when limiting analyses to men only).

**Figure 2:**
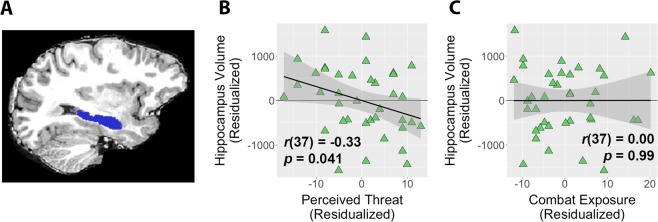
(**A**) Automatically segmented hippocampus ROI from a representative participant. (**B**) Within the post-traumatic stress symptoms (PTSS) group, higher perceived threat was correlated with smaller bilateral hippocampal volume. Hippocampal volume was not significantly correlated with combat exposure. (**C**) Plots reflect partial correlations controlling for effects of gender. Shaded areas indicate 95% confidence intervals.

Within the PTSS group, greater self-reported perceived threat while deployed was associated with significantly smaller hippocampus volume (*t*(36) = −2.09, *b* = −31.5, *p* = 0.044; Fig. 2B). Self-reported combat exposure was not significantly associated with hippocampus volume (*t*(36) = 0.01, *b* = 0.2, *p* = 0.99; Fig. 2B), and the relationship between perceived threat and hippocampus volume remained significant when controlling for combat exposure (*t*(35) = −2.06, *b* = −31.6, *p* = 0.047). This relationship was also significant when controlling for stable psychotropic medications or psychotherapy (*t*(36) = −2.04, *p* = 0.049), and was of a similar magnitude but reduced to non-significance when controlling for total intracranial volume (*t*(36) = −1.86, *p* = 0.071). In addition, regressing hippocampal volume on perceived threat while simultaneously controlling for correlated self-report measures of anxiety, depression, and worry scores showed a trend-level relationship for perceived threat (*t*(33) = −1.87, *b* = −32.4, *p* = 0.071) and no relationships for any of the other self-report indices (all |*t*s| < 0.8, all |*b*s| < 17, all *p*s > 0.4).

In contrast, there were no relationships between amygdala volume and perceived threat while deployed (*t*(36) = 0.14, *b* = 1.1, p = 0.89) or combat exposure (*t*(36) = −1.51, *b* = −11.1, p = 0.14).

### PTSD avoidance/numbing symptoms are associated with smaller hippocampal volume

**Figure 3:**
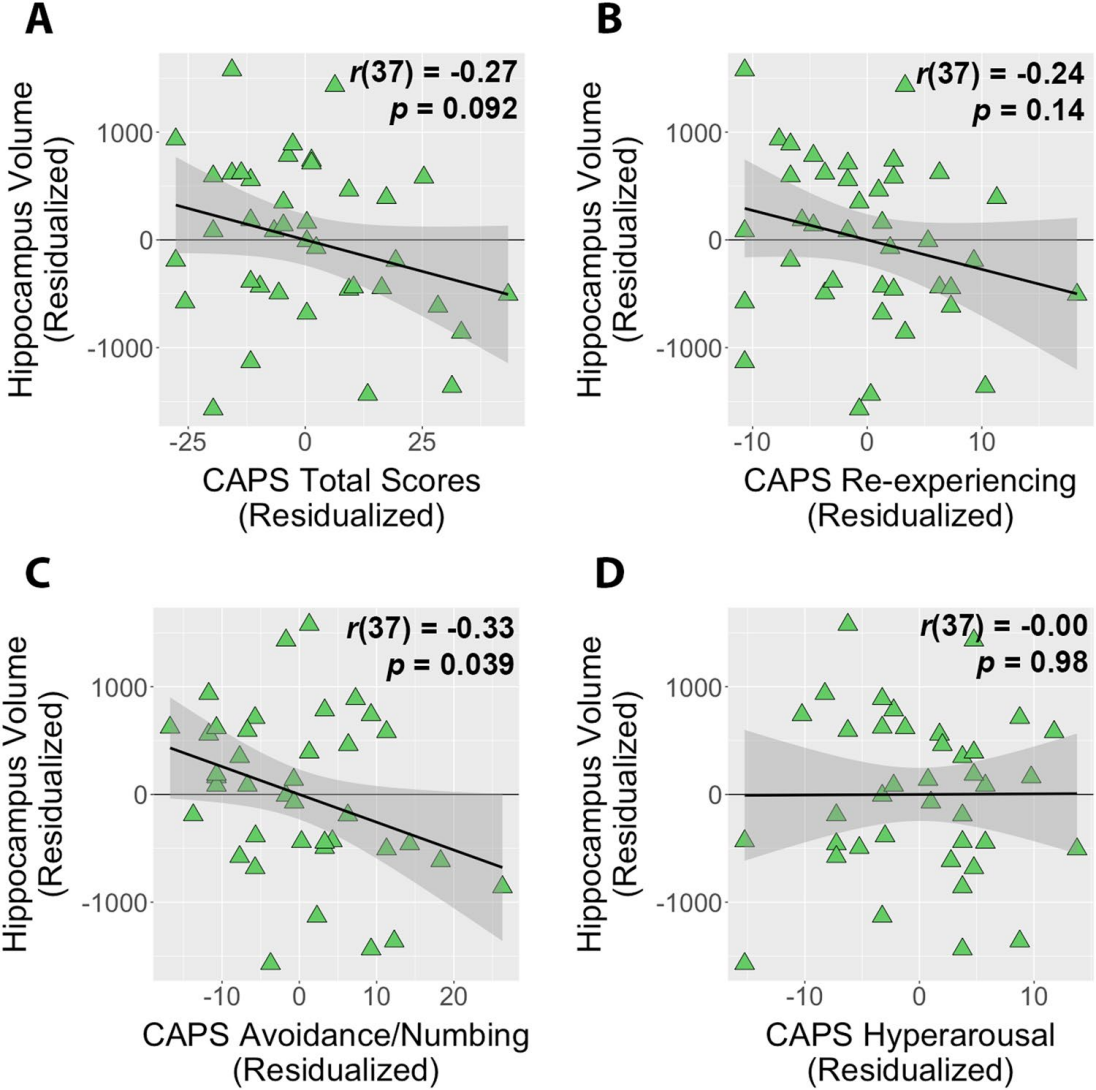
Overall PTSD symptom severity (**A**), as assessed by the Clinician-Administered PTSD Scale (CAPS), showed a trend-level negative relationship with hippocampal volume for subjects in the posttraumatic stress symptoms (PTSS) group. Hippocampal volume was inversely correlated with CAPS avoidance/numbing symptoms (**C**) in the PTSS group, and was not significantly associated with re-experiencing (**B**) or hyperarousal symptoms. (**D**) Plots reflect partial correlations controlling for effects of gender. Shaded areas indicate 95% confidence intervals.

There was a trend-level negative relationship between total PTSD symptoms on the CAPS and reduced hippocampal volume, controlling for gender (*t*(36) = −1.71, *b* = −11.7, *p* = 0.097; Fig. 3A). Examining individual symptom clusters, a significant relationship was seen for avoidance/numbing symptoms only (re-experiencing: *t*(36) = −1.51, *b* = −27.3, *p* = 0.14; avoidance/numbing: *t*(36) = −2.11, *b* = −25.8, *p* = 0.042; hyperarousal: *t*(36) = 0.03, *b* = 0.5, *p* = 0.98; Fig. 3B–D). Secondary PCL correlations with hippocampal volume were somewhat stronger and more generalized than for the CAPS (PCL total: *t*(36) = −2.71, *b* = −31.7, *p* = 0.010; re-experiencing: *t*(36) = −1.82, *b* = −61.3, *p* = 0.14; avoidance/numbing: *t*(36) = −2.40, *b* = −53.9, *p* = 0.022; hyperarousal: *t*(36) = −2.00, *b* = −70.0, *p* = 0.053; Fig. S1). There was no significant relationship between amygdala volume and total CAPS symptoms (*t*(36) = −1.29, *b* = −4.6, *p* = 0.21) or individual CAPS symptom clusters, although negative trends were observed for re-experiencing and hyperarousal symptom clusters (Fig. S2).

Individual differences in perceived threat and CAPS avoidance/numbing symptoms were each associated with significantly smaller hippocampal volume; further, perceived threat and avoidance/numbing symptoms were significantly correlated with one another. To see whether these factors explained shared or unique variance in hippocampal volume, we conducted a simultaneous linear regression of hippocampal volume on perceived threat and CAPS avoidance/numbing symptoms. Neither perceived threat (*t*(35) = −1.58, *b* = −24.4, *p* = 0.12) nor avoidance/numbing symptoms (*t*(35) = −1.61, *b* = −20.1, *p* = 0.12) accounted for unique variance in hippocampal volume; however, along with gender, these factors together accounted for 27.8% of the variance in hippocampal volume (*F*(3,35) = 5.89, *p* = 0.0023, multiple R^2^ = 0.335, adjusted R^2^ = 0.278).

### Whole-brain structural correlates of perceived threat bias and PTSD symptoms

**Figure 4:**
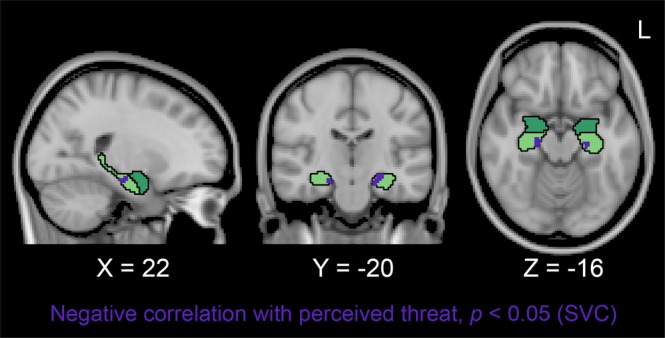
Regression of voxel-based morphometry values within the anatomically defined hippocampus (light green) and amygdala (dark green) on DRRI perceived threat. At a small-volume-corrected (SVC) threshold of *p* < 0.05, perceived threat was inversely related to local gray matter volume in the purple cluster located in bilateral anterior/mid hippocampus. Self-reported combat exposure was not significantly correlated with local gray matter volume in the hippocampus or amygdala. Non-thresholded statistical maps are available at https://neurovault.org/collections/4497.

We regressed voxelwise brain morphometry (VBM) maps on self-reported perceived threat and combat exposure within the small volume-corrected amygdala and hippocampus to confirm volumetric analyses and explore the regional specificity of our reduced hippocampal volume finding. Consistent with ROI-based volumetric analyses, perceived threat was inversely correlated with local gray matter volume in bilateral anterior/mid hippocampus (Fig. 4). Combat Exposure Scale scores were not significantly correlated with local gray matter volume in the hippocampus or amygdala. CAPS scores were not associated with local gray matter volume in the hippocampus or amygdala, and there were no clusters that survived whole-brain correction for perceived threat, combat exposure, or CAPS scores (https://neurovault.org/collections/4497/).

## Discussion

The relationship between combat experiences and maladaptive psychological outcomes has been shown to be mediated by subjective perceptions of threat^5,10,11^, but little is known about underlying neurobiological mechanisms. In a group of Operation Enduring Freedom/Operation Iraqi Freedom veterans with elevated PTSD symptoms, volumetric and VBM analyses demonstrated an inverse correlation between perceived threat and hippocampal volume while taking into account self-reported combat exposure, suggesting that this brain region may play an important role in differential threat appraisals in the aftermath of combat exposure or other traumatic events.

Previous research on perceived threat has underscored the importance of this factor in conferring risk for trauma-related psychopathology^8–11^. Although two previous studies in healthy, older adults have identified inverse correlations between hippocampal volume and the related construct of perceived stress^15,16^, we are unaware of other studies that have implicated the hippocampus in subjective threat appraisals in combat veterans or in any individuals with PTSD symptomatology. No relationship was observed for self-reported combat exposure, and a similar magnitude (though not statistically significant) effect was observed when taking into account levels of depression, anxiety, and worry symptoms, each of which was highly correlated with perceived threat scores. This suggests some degree of specificity for the hippocampus in threat appraisals, although the small sample size limits our ability to draw strong conclusions based on regression analyses with multiple covariates.

Owing to the retrospective nature of the perceived threat measure used here, it is unclear to what extent elevated levels of perceived threat reflect inflated threat appraisals *during deployment*. Such inflated appraisals would be consistent with attentional bias to threat that is observed in laboratory studies of PTSD and anxiety disorders^36^. These disorders are also associated with interpretation bias, or a tendency to interpret ambiguous information as threatening^37^. Although interpretation and attentional biases may be adaptive in unpredictable and potentially dangerous deployment contexts, such biases are less adaptive in safe, non-combat settings^20^. Continued interpretation bias in particular could function to sustain avoidant responses to trauma reminders, as captured in the avoidance/numbing PTSD symptom cluster.

Alternatively, greater perceived threat scores may reflect retrospective distortions in how certain individuals recall or report on these experiences^38^. Importantly, memories for specific traumatic events and perceived threat are malleable, and may evolve as traumatic events become more distal, particularly for individuals with elevated PTSD symptoms^39,40^. The malleability of these memories, which largely depend on the integrity of the hippocampus, makes it impossible to discern the sequence of events resulting in hippocampal alterations observed years after combat.

Our study identified cross-sectional relationships between perceived threat, hippocampal structure, and PTSD symptoms, but is unable to adjudicate between the accounts laid out above (or other alternatives). Thus, it will be important in future studies to examine these relationships longitudinally. By investigating hippocampal function and structure in relation to behavioral indices of hypervigilance and threat avoidance before, during, and after combat exposure, the causal relationship between hippocampal integrity and contextually inappropriate, maladaptive behavioral responses could be illuminated^20^. In particular, assessing perceived threat *during* deployment^7^ along with objective indices of threat exposure (based on official military records^20^) would help shed light on whether the lack of coherence between combat exposure and perceived threat (as observed here and in at least one previous report^18^) is a predisposing risk factor for PTSD, or whether this incongruency emerges in the aftermath of trauma.

This lack of coherence between perceived threat and combat exposure scores for symptomatic veterans suggests there may be value in considering a composite measure in future research on this topic, rather than examining these factors separately. It is not necessarily the case that extreme threat appraisals are maladaptive, particularly if the environment is objectively and persistently dangerous. Instead, a defining characteristic of maladaptive threat responding is incongruence between a specific context/environment and one’s response^22^. This incongruence could be further probed in future studies that investigate whether the *mismatch* between trauma exposure and subjective threat appraisals, reflected as a difference score or ratio of these measures, is a better indicator of maladaptive responding than absolute levels of perceived threat.

Further longitudinal investigations are also needed to address the long-standing question of whether reduced hippocampal volume predisposes individuals to perceive events as more threatening, or whether subjective perceptions of threat during and after deployment *contribute* to hippocampal damage, perhaps via chronic alterations to hypothalamic-pituitary-adrenal axis output. Animal research demonstrates that chronically elevated levels of glucocorticoids cause cellular damage to the hippocampus^41^ observable at the macroscopic level^42^. Human neuroimaging studies have found that basal plasma cortisol levels are inversely correlated with hippocampal volume^43,44^, and that chronic stress is associated with reduced hippocampal volume 20 years later^15^. Structural alterations of the hippocampus – whether a predisposing risk factor or a consequence of perceived stress – would have deleterious consequences for hippocampal-dependent processes such as appropriate threat contextualization^13,45^ and pattern separation ability^46^. The inability to ground threatening stimuli or fear memories in appropriate contexts may contribute to excessive avoidance of people, places, or things that bear resemblance to trauma-related stimuli, consistent with the relationship between avoidance symptoms and smaller hippocampal volume.

The relationship between PTSD symptoms and hippocampal volume reached significance only for the avoidance/numbing symptom cluster. Work from van Rooij and colleagues^47^ suggests that there are important individual differences beyond PTSD symptom severity reflected in hippocampal volume. These authors found no baseline volumetric differences between combat-exposed controls and PTSD patients who remitted following subsequent treatment, but smaller hippocampal volume in treatment-resistant PTSD patients. The authors concluded that reduced hippocampal volume is a risk factor for persistent PTSD, consistent with a classic report of smaller hippocampal volume in healthy identical twins of Vietnam veterans with PTSD^48^. Elevated perceived threat – which we found to be associated with smaller hippocampal volume – may be one factor associated with the persistence of PTSD. This suggestion is consistent with the identification here of relationships between avoidance/numbing symptoms of PTSD, perceived threat, and hippocampal volume, and previous observations of the central role of avoidance in the persistence of fear memories^49^. Notably, PTSD symptom relationships were generally more robust and less specific when using PCL instead of CAPS scores. One possible explanation for this is that self-reported PCL items may capture more generalized distress relative to the CAPS, the administration of which allows symptom reports to be validated and clarified by the interviewer. While the CAPS is the gold standard assessment for PTSD symptoms, many research studies rely on PCL scores for efficiency of administration. Parallel analysis of both measures in our study suggests they are not entirely interchangeable in a neuroscientific context. As such, future research may be warranted regarding the sensitivity and specificity of self-report vs. clinician-administered symptoms in relation to neurobiological features.

### Limitations and future directions

As alluded to above, a major limitation of this work is that our measures of perceived threat and combat exposure were both retrospective self-report measures collected on average 5 years after deployment, making them subject to response and recall biases. In addition to retrospective reporting errors, individuals with equivalent combat exposure scores may nevertheless have experienced objectively different amounts of combat trauma, as these events vary in their duration and severity. The presence of PTSD symptomatology may systematically influence retrospective reporting, leading to inflated recall of perceived threat and misreporting of combat events. Combat exposure scores in the PTSS group were positively correlated with re-experiencing symptoms, which could reflect heightened estimates of combat exposure due to flashbacks or nightmares. These factors underscore the need to reinforce cross-sectional studies such as this with longitudinal studies and the incorporation of military records (or at least concurrent self-report measures) to assess combat exposure.

Effect sizes for hippocampal relationships with perceived threat and PTSD symptoms were modest, and any attempt to apply an experiment-wide correction factor would reduce these effects to non-significance. Thus, as should be the case for all novel neuroimaging findings, these results should be interpreted with some hesitancy until confirmed by additional research. Moreover, the relatively small sample size increases the chances that the observed effect sizes could overestimate true effect sizes in the population^50^. Replication of these findings in larger and more diverse samples will be important to validate relationships between perceived threat and hippocampal structure, and to extend these results to veterans of other conflicts and non-combat trauma.

### Summary

In summary, in combat-exposed veterans with elevated PTSD symptoms, we identified a novel relationship between greater perceived threat while deployed and smaller hippocampal volume measured on average 5 years after deployment, with no such relationships observed for self-reported combat exposure or amygdala volume. These results suggest that the hippocampus is an important neural substrate for individual differences in subjective appraisals of threat, which have previously been shown to influence the development of PTSD and other mood and anxiety disorders. These results also provide an impetus for future research using longitudinal neurobiological, behavioral, and symptom-based measures to further elucidate temporal and causal relationships between elevated threat appraisal, trauma exposure, and neural structure and function.

## Supplementary information

Supplementary information

## Data Availability

The datasets generated during and/or analyzed during the current study will be made available from the corresponding author on reasonable request.
