# Supplementary material for: Elevated perceived threat is associated with reduced hippocampal volume in combat veterans
Source: Sci Rep. 2019 Oct 17;9:14888. doi: 10.1038/s41598-019-51533-x (PMC6797706; doi:10.1038/s41598-019-51533-x)
Supplement: Supplementary file 1 — Supplementary information [file 41598_2019_51533_MOESM1_ESM.pdf]

Elevated perceived threat is associated with reduced hippocampal volume in combat veterans

Supplementary Information

Daniel W. Grupe, Ph.D.*,[1,2] Benjamin A. Hushek, BS,[1] Kaley Davis, BS,[1] Andrew J. Schoen, BA,[3] Joseph Wielgosz, Ph.D.,[1,6,7] Jack B. Nitschke, Ph.D.,[4] & Richard J. Davidson, Ph.D.[1,2,4,5]

Center for Healthy Minds[1], the Waisman Laboratory for Brain Imaging and Behavior[2], and Departments of Computer Science[3], Psychiatry[4], and Psychology[5], University of Wisconsin-Madison

Sierra Pacific MIRECC, VA Palo Alto Healthcare System, Palo Alto, California[6]

Department of Psychiatry and Behavioral Sciences, Stanford University, Palo Alto, California[7]

Contents

3 **Table S1.** Correlations of perceived threat and combat exposure with PTSD symptoms.

4 **Table S2.** Correlations of perceived threat and combat exposure with symptoms of anxiety, depression, and worry

5 **Table S3**: Correlations between amygdala/hippocampal volume and perceived threat, combat exposure, and PTSD symptoms for the combat-exposed control group

6 **Table S4.** Subcortical structural volumes for the full sample and individual subgroups

7 **Figure S1**. Correlations between hippocampus volume and scores on the PTSD-Checklist – Military Version and individual symptom clusters

8 **Figure S2**. Correlations between amygdala volume and scores on the Clinician-Administered PTSD Scale and individual symptom clusters

Supplementary Tables

	Combat Exposure Scale		DRRI: Perceived Threat	
PTSS SUBGROUP (N=39)	*r*(37)	*p*	*r*(37)	*p*
CAPS total	0.17	0.29	0.22	0.17
CAPS B (Re-experiencing)	0.41	0.0089	0.14	0.41
CAPS C (Numbing/Avoidance)	-0.11	0.50	0.33	0.042
CAPS D (Hyperarousal)	0.82	0.28	0.01	0.96
CEC SUBGROUP (N=17)	*r*(15)	*p*	*r*(15)	*p*
CAPS total	-0.03	0.91	0.25	0.33
CAPS B (Re-experiencing)	0.14	0.59	0.30	0.24
CAPS C (Numbing/Avoidance)	NA	NA	NA	NA
CAPS D (Hyperarousal)	-0.08	0.77	0.14	0.61

Table S1: Pearson correlation coefficients between scores on the Combat Exposure Scale and the Perceived Threat subscale of the Deployment Risk and Resilience Inventory and symptoms of PTSD as measured by the Clinician Administered PTSD Scale (CAPS). Correlations are presented separately for the posttraumatic stress symptoms (PTSS) group (*N*=39) and the Combat-Exposed Control (CEC) group (*N*=17). Correlation coefficients could not be calculated for CAPS numbing-avoidance symptoms in the CEC group due to a lack of variability (all scores = 0).

	Combat Exposure Scale		DRRI: Perceived Threat	
**PTSS SUBGROUP (N=39)**	***r*(37)**	***p***	***r*(37)**	***p***
**Beck Depression Inventory**	-0.28	0.083	0.38	0.017
**Beck Anxiety Inventory**	-0.15	0.35	0.39	0.015
**Penn State Worry Questionnaire**	-0.15	0.36	0.47	0.0029
**CEC SUBGROUP (N=17)**	***r*(15)**	***p***	***r*(15)**	***p***
**Beck Depression Inventory**	0.18	0.49	0.39	0.12
**Beck Anxiety Inventory**	0.01	0.97	0.33	0.19
**Penn State Worry Questionnaire**	0.05	0.86	0.39	0.12

Table S2: Pearson correlation coefficients between the Combat Exposure Scale and the Perceived Threat subscale of the Deployment Risk and Resilience Inventory with symptoms of depression, anxiety, and worry. Correlations are presented separately for the posttraumatic stress symptoms (PTSS) subgroup (N=39) and the combat exposed control (CEC) subgroup (N=17).

	Hippocampus		Amygdala	
	r(15)	*p*	*r*(15)	*p*
**DRRI perceived threat**	0.13	0.61	0.05	0.85
**Combat Exposure Scale**	0.29	0.26	-0.10	0.71
**CAPS total**	0.16	0.53	0.35	0.17
**CAPS B (Re-experiencing)**	-0.27	0.30	-0.21	0.41
**CAPS C (Numbing/Avoidance)**	NA	NA	NA	NA
**CAPS D (Hyperarousal)**	0.25	0.33	0.41	0.10
**PCL total**	-0.16	0.53	0.04	0.86
**PCL B (Re-experiencing)**	0.13	0.63	0.16	0.53
**PCL C (Numbing/Avoidance)**	-0.10	0.69	-0.10	0.69
**PCL D (Hyperarousal)**	-0.29	0.26	0.08	0.76

Table S3: Pearson correlation coefficients between total hippocampus/amygdala volume and self-report/symptom measures of interest for the Combat-Exposed Control (CEC) group (*N*=17). Correlation coefficients could not be calculated for CAPS numbing-avoidance symptoms in the CEC group due to a lack of variability (all scores = 0). Notes: DRRI = Deployment Risk and Resilience Inventory; CAPS = Clinician-Administered PTSD Scale; PCL = PTSD Checklist.

	Left Hemisphere		Right Hemisphere		Bilateral	
**HIPPOCAMPAL VOLUME**	**Mean**	**SD**	**Mean**	**SD**	**Mean**	**SD**
**PTSS group (*N* = 39)**	4379	465	4388	400	8768	837
**Combat-exposed controls (*N* = 17)**	4476	425	4440	385	8916	793
**AMYGDALAR VOLUME**	**Mean**	**SD**	**Mean**	**SD**	**Mean**	**SD**
**PTSS group (*N* = 39)**	1717	200	1946	258	3663	390
**Combat-exposed controls (*N* = 17)**	1715	257	1927	242	3642	470

Table S4: Mean and standard deviation of the volume of left, right, and bilateral hippocampus and amygdala for subgroups of combat-exposed controls ($N = 17$) and the posttraumatic stress symptoms (PTSS) group ($N = 39$). Values are presented in units of mm^3.

Supplementary Figures

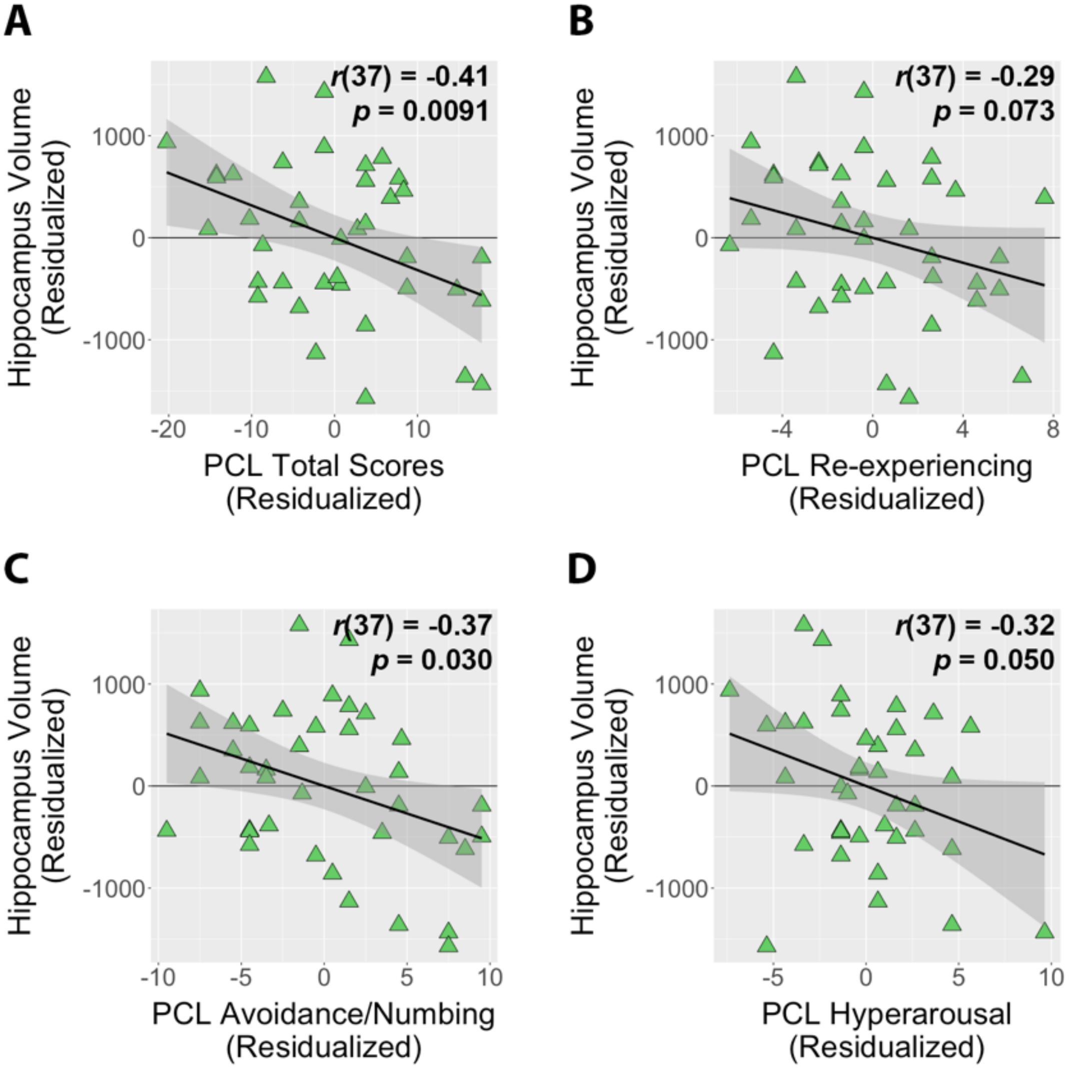

Figure S1: (A) Self-reported PTSD symptom severity on the PTSD Checklist-Military version (PCL), was significantly associated with smaller bilateral hippocampus volume for subjects in the posttraumatic stress symptoms (PTSS) group. Significant or trend-level relationships with hippocampus volume were observed for (B) re-experiencing symptoms, (C) avoidance/numbing symptoms, and (D) hyperarousal symptoms. Plots reflect partial correlations controlling for effects of gender. Shaded areas indicate 95% confidence intervals.

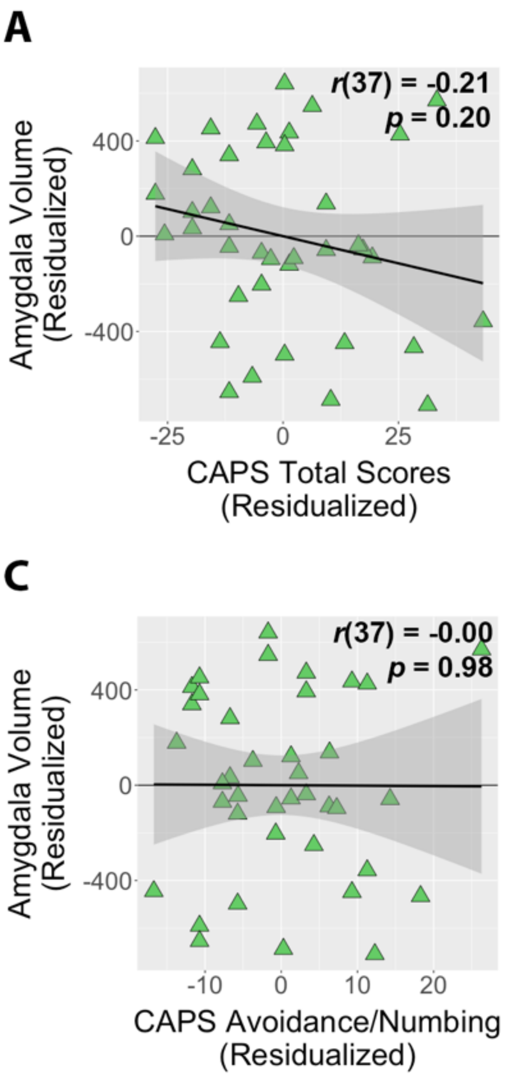

Figure S2: Overall PTSD symptom severity (A), as assessed by the Clinician-Administered PTSD Scale (CAPS), was not significantly associated with bilateral amygdala volume for subjects in the posttraumatic stress symptoms (PTSS) group. Trend-level relationships with hippocampus volume were observed for (B) re-experiencing symptoms and (D) hyperarousal symptoms, but not (C) avoidance/numbing symptoms. Plots reflect partial correlations controlling for effects of gender. Shaded areas indicate 95% confidence intervals.
